# Long-term results of mitral valve repair using leaflet resection versus chordae replacement

**DOI:** 10.1016/j.xjtc.2026.102292

**Published:** 2026-02-25

**Authors:** Miriam Lang, Leon Schmidt, Lucas Fernando Sarmiento Rios, Nina Feirer, Andrea Amabile, Arnar Geirsson, Tobias Rheude, Bernhard Voss, Markus Krane, Keti Vitanova

**Affiliations:** a Department of Cardiovascular Surgery, TUM University Hospital German Heart Center, TUM School of Medicine & Health, Technical University of Munich, Munich, Germany; b Division of Cardiac Surgery, Department of Cardiothoracic Surgery, University of Pittsburgh, Pittsburgh, Pa; c Section of Cardiac Surgery, Department of Surgery, Columbia University, New York, NY; d Department of Cardiology, TUM University Hospital German Heart Center, TUM School of Medicine & Health, Technical University of Munich, Munich, Germany; e DZHK (German Center for Cardiovascular Research) – Partner Site Munich Heart Alliance, Munich, Germany

**Keywords:** mitral valve, mitral valve repair, long-term outcomes, chordae replacement, leaflet resection

## Abstract

**Objective:**

The available data on long-term durability of mitral valve repair using chordae replacement versus leaflet resection for degenerative mitral regurgitation (MR) is contradictory. Although some studies report equal long-term durability, others demonstrate worse durability for chordae replacement.

**Methods:**

All consecutive patients receiving mitral valve repair using chordae replacement versus leaflet resection for degenerative MR from January 2003 to December 2010 at a high-volume single center were analyzed. Long-term survival, cumulative incidence of reoperation, and recurrent MR as well as echocardiographic follow-up were investigated.

**Results:**

In total, 658 patients were included. Median follow-up was 13.6 years (interquartile range, 9.5-16.0 years). Chordae replacement and leaflet resection were performed in 72.5% (n = 477) and 24.2% (n = 159) of cases, respectively. At unadjusted analysis, cases of leaflet resection tended to have worse survival when compared with cases of chordae replacement (66.3% ± 4.1% vs 77.2% ± 2.3% at 15 years; *P* = .053). Patients with chordae replacement had a significantly higher risk for recurrent MR (33.4% ± 3.6% vs 12.1% ± 3.6% at 15 years; *P* < .001) and reoperation (19.1% ± 2.4% vs 7.1% ± 2.6% at 15 years; *P* = .013) than patients with leaflet resection. The risk remained significant even after excluding all patients with Barlow's disease and endocarditis. Risk factors for recurrent MR were chordae replacement (hazard ratio, 2.218; 95% CI, 1.237-3.979; *P* = .008), endocarditis (hazard ratio, 3.382; 95% CI, 1.676-6.825; *P* < .001), and MR >I at discharge (hazard ratio, 5.602; 95% CI, 3.009-10.441; *P* < .001).

**Conclusions:**

Mitral valve repair using chordae replacement was associated with slightly higher survival rates and significantly higher cumulative incidence of MR recurrence and reoperation when compared with mitral valve repair using leaflet resection.

## Graphical Abstract

**Figure:**
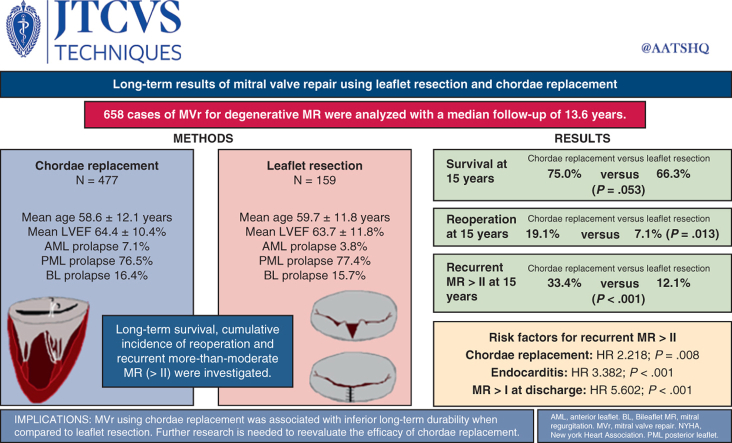

**Figure:**
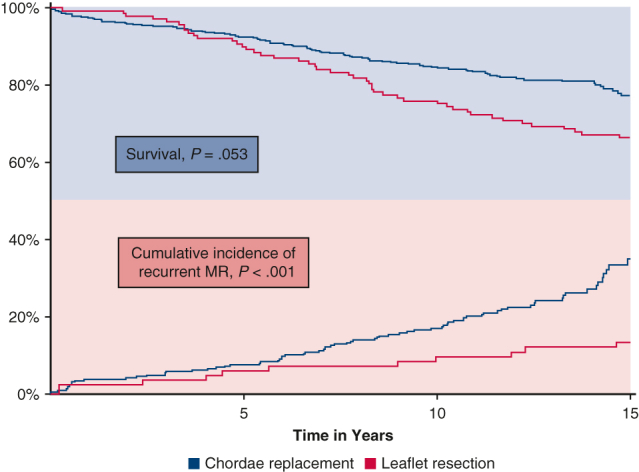
Survival is shown in yellow. Cumulative incidence of recurrent MR >II is shown in green.

**Central Message**

Mitral valve repair using chordae replacement was associated with inferior long-term durability in patients with degenerative mitral regurgitation when compared with leaflet resection.

**Perspective**

Chordae replacement compared with leaflet resection for repair of degenerative mitral valve regurgitation was associated with increased cumulative incidence of recurrent more-than-moderate mitral regurgitation. Further research is needed to re-evaluate the efficacy of chordae replacement and to investigate the discrepancy between both techniques.

Mitral valve repair (MVr) is the preferred surgical treatment for degenerative mitral regurgitation (DMR) according to both the European Society of Cardiology/European Association for Cardio-Thoracic Surgery and the American College of Cardiology/American Heart Association guidelines on management of valvular heart disease.^1^^,^^2^ There is a broad spectrum of repair techniques of the mitral valve (MV); however, the most frequently used techniques are leaflet resection and chordae replacement. Multiple studies have compared both techniques and have found equally low mortality, but data on durability remains less definitive.^3^, ^4^, ^5^, ^6^, ^7^, ^8^, ^9^

On 1 hand, there are studies demonstrating comparable outcome for both techniques.^3^, ^4^, ^5^^,^^9^ During 2008, a prospective randomized trial including patients with posterior mitral leaflet (PML) prolapse reported comparable results for both techniques at 1 year.^9^ Another study analyzed patients with left ventricular (LV) dilatation and severe DMR due to PML prolapse and demonstrated comparable rates of recurrent more-than-moderate mitral regurgitation (MR) at 10 years.^5^ However, not only PML prolapse was investigated: 1 study analyzing any leaflet prolapse demonstrated comparable reoperation rates after 10 years,^3^ and another study comparing both MVr techniques in patients with Barlow's disease could not identify any difference in reoperation rates or MR recurrence.^4^

On the other hand, some more recent studies demonstrated worse long-term durability of chordae replacement when compared with leaflet resection.^6^^,^^10^^,^^7^ Ogami and colleagues^6^ compared both techniques in patients with PML prolapse and reported a higher cumulative incidence of MV reoperation after chordae replacement at 5 years. Another study also demonstrated higher probability of recurrent MR after chordae replacement, despite similar survival and reoperation rates in both groups.^10^ One study including any leaflet prolapse reported significantly higher incidence of reoperation and recurrent severe MR at 10 years after MVr using chordae replacement.^7^ Hence, the data are contradictory, and the underlying causes for the widely varying results are unknown. The aim of this study is to analyze the long-term outcomes of MVr using chordae replacement and leaflet resection at a high volume-center.

## Material and Methods

The study protocol was approved by the Research Ethics Committee of the Technical University Munich (Study No.: 2024-183-S-CB; date of approval, April 9, 2024). Written informed consent was waived due to the retrospective design of this study.

All consecutive patients undergoing MVr for DMR at the German Heart Center Munich from January 2003 to December 2010 were included. Exclusion criteria were any concomitant cardiac procedures (except for the maze procedure or procedures on the atrial septum), isolated annuloplasty for MVr, previous cardiac surgery and age younger than 18 years. We included data on operative protocols, in-hospital stay, echocardiographic studies and follow-up data. When follow-up data were not available in the database, it was collected from referring cardiologists with informed consent of the patients. The degree of MR was classified as none/trivial (0), mild (I), moderate (II), moderate-to-severe (III), or severe (IV), as assessed by means of color Doppler flow mapping.^11^ Follow-up data included active symptoms, concomitant diseases, and need for reoperation on the MV. When survival data were not available in our database, it was systematically verified by telephone interview or written correspondence with the patient, their relatives, their referring cardiologist, or their general practitioner. Follow-up regarding survival, reoperation, and recurrent more-than-moderate MR was complete for 93.6%, 69.5%, and 58.7% of patients, respectively. Echocardiographic follow-up included all the latest available echocardiographic data. The long-term results of MVr in a highly selected cohort of repairs using solely chordae replacement were previously published by our group.^12^ In contrast, the present analysis investigates all MVrs using resection techniques and chordae replacement. The aim of this study is to compare long-term outcomes according to the used repair technique. We investigated long-term survival, cumulative incidence of reoperation on the MV, and of recurrent more-than-moderate MR, echocardiographic follow-up data, as well as risk factors for death and MR recurrence.

### Operative Technique

Operation was usually performed via median sternotomy or anterolateral thoracotomy. Two cases were performed via partial sternotomy. Cannulation for cardiopulmonary bypass (CPB) depended on the method of thoracotomy. For all techniques, CPB was established in moderate hypothermia and cardioplegic arrest was achieved using a crystalloid solution. MVr was performed by 5 surgeons with at least 10 years of experience. Annuloplasty was performed in all cases using either a ring or a band, except for 2 cases with extensive calcifications of the MV annulus. Patients received either chordae replacement (neochord group) or leaflet resection (resection group) as repair technique, only rarely (n = 22) a combination of both techniques was necessary. Chordae replacement was performed using 4-0 expanded polytetrafluoroethylene sutures as described previously by our group.^13^ Leaflet resection was performed either as quadrangular or triangular resection as described by Carpentier.^14^ Additionally, leaflet edge-to-edge repair was performed as needed. The operative result was evaluated intraoperatively in all cases using transesophageal echocardiography. In case of intraoperative residual MR >I, the repair was improved with a subsequent CPB run.

### Statistical Analysis

Statistical analysis was performed using SPSS version 29.0 (IBM-SPSS Inc) and NCSS 2025 (NCSS, LLC). Continuous variables were reported as mean ± SD, except stated otherwise. Comparisons of variables at baseline, operative characteristics, and follow-up data were performed using *t* test for continuous variables and χ^2^ test for categorical variables. For the statistical analysis, all patients receiving a combination of chordae replacement and resection techniques were excluded. Kaplan-Meier analysis was used for estimation of survival and significance was assessed using the log-rank test. The cumulative incidence of recurrent MR >II and reoperation on the MV was calculated with death as the competing risk, significance was assessed using the Gray test. A subgroup analysis of the competing risk analysis was performed excluding all patients with endocarditis and Barlow's disease. Patients with missing data regarding reoperation and recurrent significant MR were excluded from the respective competing risk analysis. Univariable and multivariable Cox regression analysis was used to determine independent risk factors of mortality and recurrent MR. Covariables included in the calculation and results of univariable Cox regression analysis are summarized in Tables E1 and E2. Covariables with a *P* value of < .1 in univariable analysis, were included in multivariable analysis. Hazard ratios and 95% CI per covariable were estimated.

## Results

### Patient Data

**Figure 1:**
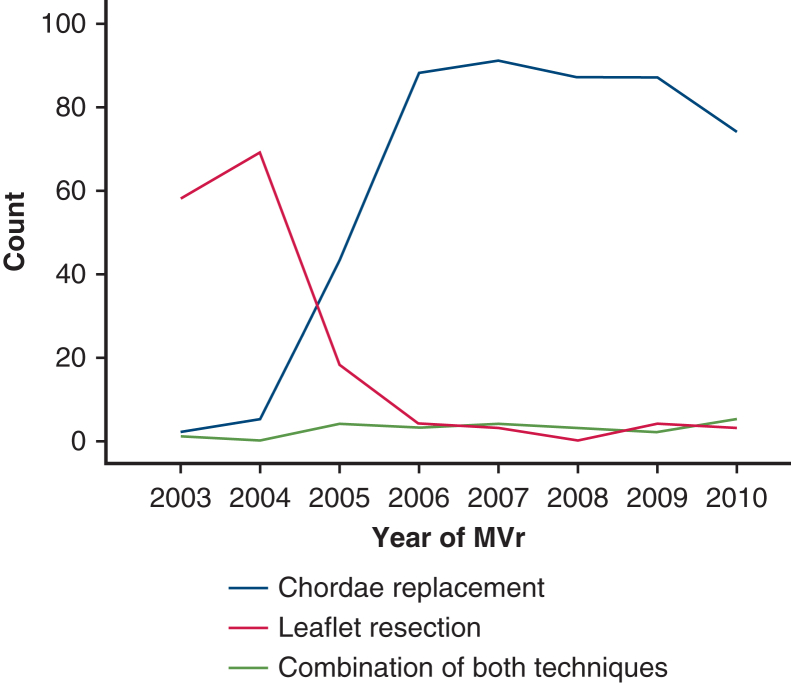
Timeline of the frequency of mitral valve repair (*MVr*) techniques during the study period.

From January 2003 to December 2010, 658 patients underwent MVr using resection techniques and/or chordae replacement for DMR at the German Heart Center Munich. Chordae replacement was performed in 72.4% (n = 477) and leaflet resection was performed in 24.1% (n = 159). In 3.3% (n = 22) a combination of both techniques was used. During the study period a difference in the frequency of the respective repair methods was observed: leaflet resection was predominantly performed from 2003-2004, whereas chordae replacement was more frequent from 2005-2010 (Figure 1). The baseline characteristics are summarized in Table 1. Patients in the neochord group were more likely to experience arterial hypertension. Patients in the resection group were more likely to present with reduced left ventricular ejection fraction (LVEF) <55% and diabetes. Operative characteristics are summarized in Table 2. Right lateral minithoracotomy was the most common approach in both groups but was significantly more frequent in the neochord group. Barlow's disease was significantly more common in the neochord group. Patients with acute endocarditis at baseline more frequently received leaflet resection. Mean size of annuloplasty rings or bands was significantly larger when chordae replacement was performed. Residual MR >I at discharge was slightly more common in the neochord group than in the resection group. Perioperative complications were low in both groups and are summarized in Table E3.

**Table 1:** Baseline characteristics

Variable	Total cohort	Chordae replacement	Leaflet resection	*P* value
Female sex	186 ± 28.3	132 ± 27.7	50 ± 31.4	.362
Age (y)	58.6 ± 12.1	58.6 ± 12.1	59.7 ± 11.8	.282
Symptoms according to NYHA class				
NYHA I	17 (2.6)	14 (3.0)	3 (1.9)	.463
NYHA II	48 (7.4)	33 (7.0)	12 (7.5)	.824
NYHA III	507 (77.9)	356 (75.7)	135 (84.9)	.016
NYHA IV	79 (12.1)	67 (14.3)	9 (5.7)	.004
Level of MR				
MR I	2 (0.3)	2 (0.4)	0	.418
MR II	46 (7.5)	36 (8.0)	10 (6.8)	.632
MR III	442 (71.8)	323 (71.9)	105 (71.4)	.905
MR IV	126 (20.5)	88 (19.6)	32 (21.8)	.569
LVEF <55%	84 (13.1)	52 (11.2)	28 (17.9)	.031
Atrial fibrillation	151 (23.9)	104 (23.1)	45 (28.5)	.173
Diabetes	27 (4.1)	15 (3.2)	12 (7.5)	.018
Arterial hypertension	317 (49.6)	247 (53.8)	60 (38.0)	<.001
Hyperlipidemia	113 (17.7)	73 (15.9)	33 (20.9)	.152
Pulmonary arterial hypertension	143 (22.4)	105 (23.0)	34 (21.5)	.706

Values are presented as mean ± SD or n (%). *NYHA*, New York Heart Association; *MR*, Mitral regurgitation; *LVEF*, left ventricular ejection fraction.

**Table 2:** Operative characteristics

Variable	Total cohort	Chordae replacement	Leaflet resection	*P* value
Approach				
Median sternotomy	174 (26.4)	108 (22.6)	62 (39.0)	<.001
Right lateral thoracotomy	482 (73.3)	368 (77.1)	96 (60.4)	<.001
Partial sternotomy	2 (0.3)	1 (0.2)	1 (0.6)	.413
Acute endocarditis	25 (3.8)	6 (1.3)	16 (10.1)	<.001
Barlow's disease	99 (15.1)	74 (15.5)	14 (8.9)	.036
Prolapse localization				
AML prolapse	42 (6.4)	34 (7.1)	6 (3.8)	.131
PML prolapse	495 (75.2)	365 (76.5)	123 (77.4)	.828
BL prolapse	115 (17.5)	78 (16.4)	25 (15.7)	.852
Quadrangular resection	156 (23.7)	0	145 (91.2)	NA
Triangular resection	30 (4.6)	0	18 (11.3)	NA
Number of neochords	2.73± 1.1	2.74 ± 1.1	0	NA
Annuloplasty size (mm)	33.1 ± 3.2	33.7 ± 3.2	30.9 ± 2.2	<.001
Edge-to-edge repair	29 (4.4)	15 (3.1)	12 (7.5)	.017
PFO closure	25 (3.8)	21 (4.4)	4 (2.5)	.289
Maze procedure	39 (5.9)	27 (5.7)	11 (6.9)	.562
LAA occlusion	17 (2.6)	13 (2.7)	4 (2.5)	.887
Crossclamp time (min)	85.5 ± 25.1	84.6 ± 23.4	84.0 ± 24.0	.786
MR >I at discharge	22 (3.6)	19 (4.3)	2 (1.4)	.095

Values are presented as n (%) or mean ± SD, except stated otherwise. *AML*, Anterior mitral leaflet; *PML*, posterior mitral leaflet; *BL*, bileaflet; *NA*, not applicable; *PFO*, persistent foramen ovale; *LAA*, left atrial appendage.

### Survival

**Figure 2:**
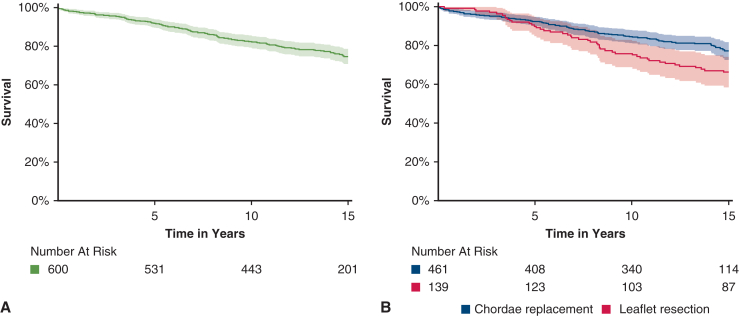
Kaplan-Meier survival curves with 95% confidence limits. A, Survival of the total cohort. B, Survival of patients with chordae replacement versus leaflet resection.

The median follow-up was 13.6 years (interquartile range, 9.5-16.0). The overall mortality was 26.5% (n = 163). Cumulative mortality was more frequent in the resection group (43.9% [n = 61]) when compared with the neochord group (21.5% [n = 99]) over the study period. Multivariable Cox regression identified age, atrial fibrillation, diabetes, reduced LVEF <55% and MR >I at discharge as independent risk factors for mortality. Right lateral thoracotomy was associated with a reduced risk for death (Table 3). Overall, survival at 5, 10, and 15 years was 92.2% ± 1.1%, 82.6% ± 1.6%, and 75.0% ± 1.9% (Figure 2, *A*). Survival at 5, 10, and 15 years was 90.6% ± 2.5%, 75.9% ± 3.7%, and 66.3% ± 4.1% in the resection group; and 92.5% ± 1.3%, 84.6% ± 1.7%, and 77.2% ± 2.3% in the neochord group, respectively (*P* = .053) (Figure 2, *B*).

**Table 3:** Independent risk factors as identified by multivariable Cox regression

Parameter	Hazard ratio	95% CI	*P* value
Risk factors associated with mortality			
Female sex	1.259	0.857-1.849	.241
Age at baseline	1.074	1.049-1.099	<.001
Atrial fibrillation	1.451	1.005-2.096	.047
Diabetes	2.313	1.234-4.335	.009
Pulmonary arterial hypertension	1.040	0.721-1.500	.834
Arterial hypertension	1.341	0.943-1.905	.102
LVEF <55%	2.125	1.376-3.282	.001
Right lateral thoracotomy	0.445	0.301-0.658	<.001
Leaflet resection	1.086	0.748-1.576	.665
MR >I at discharge	4.217	2.053-8.662	<.001
Risk factors associated with recurrent more-than-moderate MR			
Chordae replacement	2.218	1.237-3.979	.008
Endocarditis	3.382	1.676-6.825	<.001
MR >I at discharge	5.605	3.009-10.441	<.001

*LVEF*, Left ventricular ejection fraction; *MR*, mitral regurgitation.

### Recurrent MR

**Figure 3:**
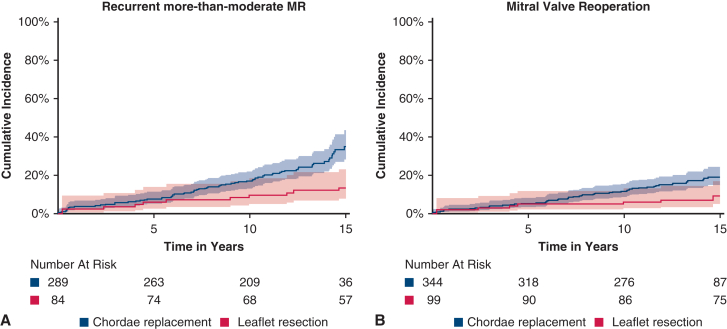
Comparison of cumulative incidence between patients with chordae replacement and leaflet resection with death as the competing risk. Curves are shown with 95% confidence limits. A, Cumulative incidence of recurrent more-than-moderate mitral regurgitation (*MR*). B, Cumulative incidence of mitral valve reoperation.

**Figure E1:**
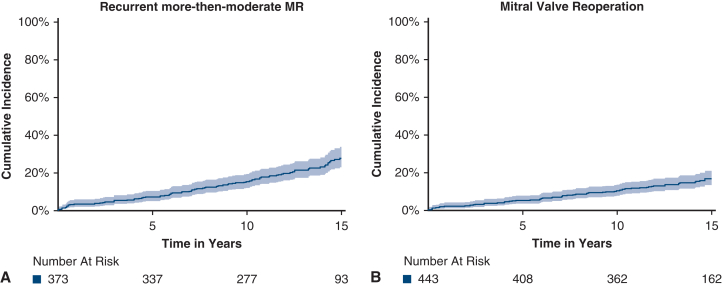
Cumulative incidence plots with death as the competing risk and 95% confidence limits. A, Cumulative incidence of recurrent more-than-moderate mitral regurgitation (*MR*). B, Cumulative incidence of reoperation.

Recurrent MR >II was observed in 26.7% (n = 103). The independent risk factors associated with recurrent MR were chordae replacement, endocarditis, and MR >I at discharge (Table 3). The cumulative incidence of recurrent MR >II in the total cohort was 7.0% ± 1.3%, 14.7% ± 1.8%, and 27.0% ± 2.6% at 5, 10, and 15 years, respectively (Figure E1, *A*). Patients of the resection group had a cumulative incidence of recurrent MR >II of 6.0% ± 2.6%, 8.4% ± 3.0%, and 12.1% ± 3.6% at 5, 10, and 15 years each. The cumulative incidence of recurrent MR >II in the neochord group was 6.9% ± 1.5%, 16.5% ± 2.2%, and 33.4% ± 3.6% at 5, 10, and 15 years, respectively (resection vs neochord group, *P* < .001) (Figure 3, *A*). After the exclusion of patients with endocarditis and Barlow's disease, the cumulative incidence of recurrent MR >II remained significantly higher in the neochord group than in the resection group (32.7% ± 4.1% vs 10.1% ± 3.6% at 15 years; *P* < .001) (Table E4).

### Reoperation

**Figure E2:**
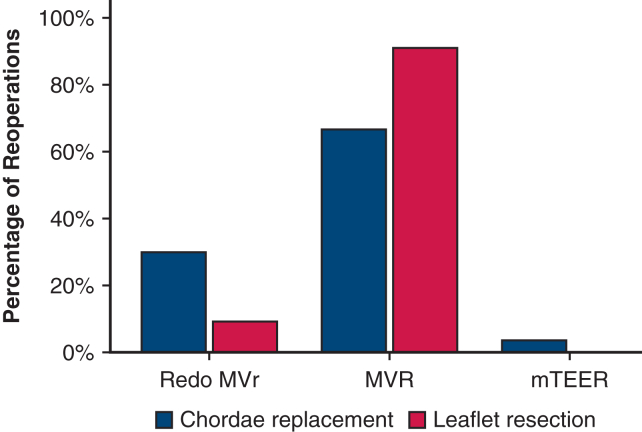
Methods of reoperation after mitral valve repair (*MVr*) using chordae replacement or leaflet resection. *mTEER*, Mitral transcatheter edge-to-edge repair; *MVR*, mitral valve replacement.

Reoperation on the MV was necessary in 16.2% (n = 74). MV replacement was the most frequent method of reoperation with 66.7% (n = 38) of patients after chordae replacement and 90.9% (n = 10) of patients after leaflet resection (*P* = .106). Redo MVr was more often feasible in the neochord group (29.8% [n = 38]) when compared with the resection group (9.1% [n = 1]; *P* = .154). Mitral transcatheter edge-to-edge repair was performed in 2 cases of reoperation after chordae replacement (Figure E2). In total, the cumulative incidence of reoperation was 5.3% ± 1.1%, 10.0% ± 1.4%, and 16.4% ± 1.9% at 5, 10, and 15 years each (Figure E1, *B*). The cumulative incidence of reoperation was significantly lower in the resection group with 5.1% ± 2.2%, 5.1% ± 2.2%, and 7.1% ± 2.6%, than in the neochord group with 5.0% ± 1.2%, 11.3% ± 1.7%, and 19.1% ± 2.4% at 5, 10, and 15 years, respectively (*P* = .013) (Figure 3, *B*). After the exclusion of patients with endocarditis and Barlow's disease, the cumulative incidence of reoperation was 18.2% ± 2.6% in the neochord group and 6.4% ± 3.8% in the resection group at 15 years (*P* = .017) (Table E5).

### Echocardiography Follow-up

**Figure 4:**
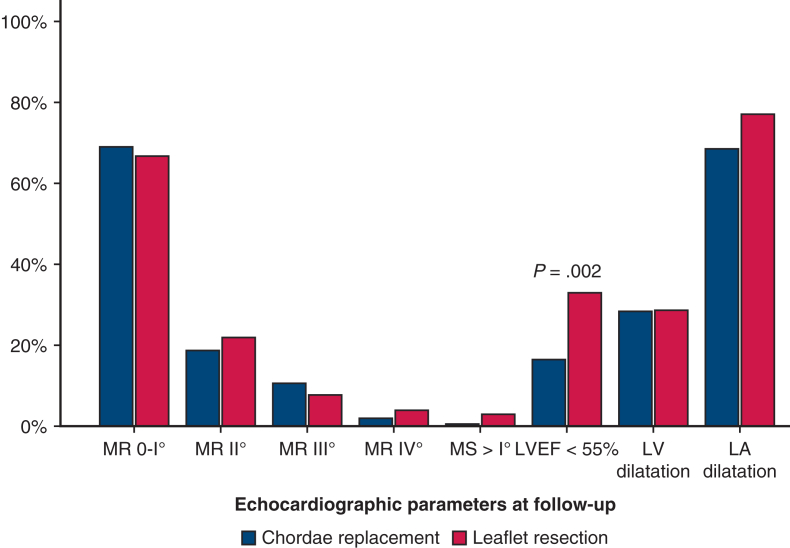
Comparison of echocardiographic follow-up data after mitral valve repair using chordae replacement and leaflet resection. *MR*, Mitral regurgitation; *MS*, mitral stenosis; *LVEF*, left ventricular ejection fraction; *LV*, left ventricle; *LA*, left atrium.

Data is summarized in Figure 4 and Table E6. The proportion of patients with LVEF <55% was higher among cases with leaflet resection. Accordingly, mean LVEF tended to be lower after MVr using leaflet resection with 55.5% ± 11.6% when compared with chordae replacement (58.9% ± 8.5%; *P* = .051).

### Comment

This retrospective study analyzed 658 patients after MVr using chordae replacement versus leaflet resection techniques. Comparison of leaflet resection and chordae replacement for MVr showed that chordae replacement carries a significantly higher risk of recurrent MR and MV reoperation. Accordingly, MVr using chordae replacement was associated with recurrent more-than-moderate MR in Cox regression analysis. Other than that, MR >I at discharge and endocarditis were also identified as risk factors for recurrent more-than-moderate MR. Age, atrial fibrillation, diabetes, reduced LVEF <55%, and MR >I at discharge were identified as independent risk factors for death.

### Survival

The cumulative mortality was higher in cases of leaflet resection. This might be explained by the fact that those patients were operated on during the earlier years of the study period. Hence, were older at follow-up. Another explanation could be a worse state of health at operation when compared with the patients in the neochord group. Because diabetes and reduced LVEF <55% at baseline, which were risk factors associated with mortality, were more common at baseline in the resection group. Another reason for worse survival might be a significantly higher percentage of reduced LVEF <55% at echocardiographic follow-up in the resection group. A study comparing resection techniques and chordae replacement in a cohort of patients with LV dilatation showed a higher reduction of LV end-diastolic diameter and a better LVEF at 6 years after chordae replacement.^5^ Although we did not observe any differences in LV diameters at follow-up, we did observe a lower mean LVEF at follow-up in the resection group despite a comparable mean LVEF at baseline.

### Recurrent MR and MV Reoperation

Patients after MVr using chordae replacement had a significantly higher cumulative incidence of recurrent MR and MV reoperation in long-term follow-up when compared with resection techniques. Additionally, chordae replacement was identified as an independent risk factor for recurrent MR. Other studies have also demonstrated worse durability for chordae replacement in comparison to leaflet resection.^6^^,^^7^^,^^10^ In a recent study by Kakuta and colleagues^8^ chordae replacement was also identified as risk factor for recurrent more-than-moderate MR. One reason might be technical failure like rupture of neochords, or LV remodeling after MVr leading to length mismatch of the implanted neochords.^5^^,^^15^ In an ex vivo heart stimulation investigating neochord length and its influence on MV hemodynamics even small differences of a few millimeters in neochord length significantly altered valvular biomechanics.^16^ This underscores the significance of ensuring the optimal length of neochords. However, a multitude of suturing techniques for the implantation of neochords exist^13^^,^^17^, ^18^, ^19^ and the number of neochords is not standardized. Consequently, techniques vary considerably between institutions. This heterogeneity in techniques may contribute to the ambiguity observed in the published data. Another reason for worse durability of MVr using chordae replacement might be disease progression because MR >I at discharge was a risk factor for recurrent MR and tended to be more common in patients after chordae replacement. In fact, MR >I at discharge is proven to be the most powerful predictor of recurrent MR.^20^ The higher rates of residual MR in the neochord group might be explained by a slightly higher percentage of Barlow's disease and anterior prolapse at baseline. However, when excluding patients with Barlow's disease, we could not observe a difference in cumulative incidence of recurrent MR. Finally, the exact reason for worse durability after MVr using chordae replacement in this study remains unclear. DMR is a highly variable disease and surgeons performing MVr should not rigidly adhere to 1 method but rather utilize the full range of repair techniques available as required.

### Limitations

The study is limited due to its retrospective, and single-center design. In addition, follow-up data regarding reoperation and echocardiographic data were not as complete as for survival. The percentage of deaths was higher among patients with leaflet resection and, because the index operation was up to 22 years ago, the information whether reoperation was performed or recurrent MR occurred before death was partially incomplete. Those patients were excluded in the competing risk analysis; nevertheless, this might add substantial bias to our analysis. In addition, time bias is present in this study as leaflet resection was more frequent in the earlier study period and was then largely replaced by chordae replacement. Echocardiographic follow-up was performed by multiple referring cardiologists with no existing protocol and no predefined or unified interval. The technique used for MVr was subject to the surgeon's discretion, hence selection bias cannot be ruled out.

## Conclusions

Leaflet resection was associated with slightly lower survival rates and chordae replacement was associated with a significantly higher cumulative incidence of recurrent significant MR and MV reoperation. In addition, chordae replacement was identified as an independent risk factor for MR recurrence.

### Webcast

You can watch a Webcast of this AATS meeting presentation by going to: https://www.aats.org/resources/long-term-results-of-mitral-va-11326.

**Figure:**
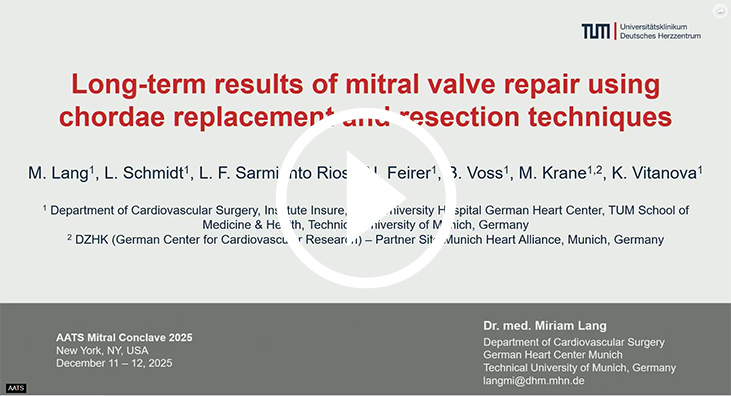

## Conflict of Interest Statement

Dr Vitanova is a consultant for Medtronic and Astra Zeneca and has received speaker fees from Medtronic, Edwards, and AtriCure. Dr Krane is a physician proctor and a member of the medical advisory board for Sanamedi and Edwards and has received speakers' honoraria from Edwards, AtriCure, CytoSorbents, and Novo Nordisk. Dr Geirsson has received consulting fees from Edwards Lifescience and Medtronic. Dr Voss is a consultant for Medtronic. All other authors have no conflicts of interest.

The *Journal* policy requires editors and reviewers to disclose conflicts of interest and to decline handling or reviewing manuscripts for which they may have a conflict of interest. The editors and reviewers of this article have no conflicts of interest.

## Figures and Tables

**Table E1:** Independent risk factors associated with mortality identified by univariable Cox regression

Parameter	Hazard ratio	95% CI	*P* value
Female sex	1.427	1.027-1.984	.034
Age at baseline	1.105	1.085-1.125	<.001
Atrial fibrillation	3.140	2.282-4.320	<.001
Diabetes	3.094	1.750-5.471	<.001
Pulmonary arterial hypertension	1.533	1.086-2.164	.015
Arterial hypertension	1.640	1.188-2.265	.003
Hyperlipidemia	1.199	0.808-1.780	.368
LVEF <55%	1.896	1.285-2.798	.001
Endocarditis	0.740	0.327-1.674	.470
Right lateral thoracotomy	0.248	0.181-0.339	<.001
Leaflet resection	1.400	0.995-1.972	.054
MR >I at discharge	2.391	1.215-4.706	.012

*LVEF*, Left ventricular ejection fraction; *MR*, mitral regurgitation.

**Table E2:** Independent risk factors of recurrent more-than-moderate mitral regurgitation (MR) identified by univariable Cox regression

Parameter	Hazard ratio	95% CI	*P* value
Right lateral thoracotomy	0.974	0.584-1.623	.918
Chordae replacement	2.586	1.465-4.565	.001
Endocarditis	2.641	1.371-5.087	.004
Barlow's disease	1.334	0.767-2.318	.307
AML prolapse	1.555	0.719-3.361	.262
PML prolapse	0.796	0.499-1.270	.339
BL prolapse	1.038	0.599-1.798	.895
MR >I at discharge	5.938	3.216-10.961	<.001
Second CPB run	1.468	0.461-4.673	.516

*AML*, Anterior mitral leaflet; *PML*, posterior mitral leaflet; *BL*, bileaflet; *CPB*, cardiopulmonary bypass.

**Table E3:** Complications

Variable	Total cohort	Chordae replacement	Leaflet resection	*P* value
Rethoracotomy for bleeding	14 (2.1)	9 (1.9)	5 (3.2)	.337
Pericardiotomy for pericardial effusion/tamponade	6 (0.9)	2 (0.4)	3 (1.9)	.067
Pacemaker implantation	1 (0.2)	1 (0.2)	0	.566
Cerebral ischemia/bleeding	3 (0.5)	2 (0.4)	1 (0.6)	.730
Myocardial infarction	1 (0.2)	1 (0.2)	0	.563
Abdominal bleeding	3 (0.5)	3 (0.6)	0	.319
Aortic dissection	1 (0.2)	1 (0.2)	0	.566
Lymphocele	6 (0.9)	2 (0.4)	3 (1.9)	.067
Thoracic wall hernia	2 (0.3)	2 (0.4)	0	.416
Femoral bleeding	2 (0.3)	2 (0.4)	0	.416
Renal insufficiency	3 (0.5)	3 (0.6)	0	.319
Atrioventricular groove disruption	2 (0.3)	2 (0.4)	0	.416

**Table E4:** Cumulative incidence of recurrent mitral valve regurgitation with death as competing risk: Subanalysis excluding patients with endocarditis and Barlow's disease (*P* value < .001)

Variable	Cumulative incidence (%)	SE (%)	95% CI	
			Lower limit	Upper limit
Mitral valve repair with chordae replacement				
5 y	7.7	1.7	4.9	12.0
10 y	14.3	2.3	10.4	19.5
15 y	32.7	4.1	25.6	41.8
Mitral valve repair with leaflet resection				
5 y	4.3	2.4	1.4	13.0
10 y	7.2	3.1	3.1	16.7
15 y	10.1	3.6	5.0	20.4

**Table E5:** Cumulative incidence of mitral valve reoperation with death as competing risk: Subanalysis excluding patients with endocarditis and Barlow's disease (*P* value = .017)

Variable	Cumulative incidence (%)	SE (%)	95% CI	
			Lower limit	Upper limit
Mitral valve repair with chordae replacement				
5 y	5.1	1.3	3.0	8.4
10 y	9.9	1.8	6.9	14.1
15 y	18.2	2.6	13.8	24.1
Mitral valve repair with leaflet resection				
5 y	2.6	2.2	0.7	10.1
10 y	3.9	2.8	1.3	11.7
15 y	6.4	3.8	2.8	15.0

**Table E6:** Echocardiographic follow-up data.

Variable	Total cohort	Chordae replacement	Leaflet resection	*P* value
MR I	239 (68.7)	178 (69.0)	52 (66.7)	.699
MR II	67 (19.3)	48 (18.6)	17 (21.8)	.532
MR III	34 (9.8)	27 (10.5)	6 (7.7)	.471
MR IV	8 (2.3)	5 (1.9)	3 (3.9)	.333
MS >I	4 (1.2)	1 (0.4)	2 (2.7)	.062
LVEF (%)	58.0 ± 9.4	58.9 ± 8.5	55.5 ± 11.6	.051
LVEF <55%	63 (20.7)	36 (16.4)	24 (32.9)	.002
LVEDD (mm)	52.8 ± 7.8	52.6 ± 6.8	52.5 ± 9.8	.966
LV dilatation	81 (28.9)	58 (28.3)	18 (28.6)	.966
LA dilatation	198 (71.2)	141 (68.4)	47 (77.1)	.196
sPAP	30.7 (9.5)	30.6 (7.8)	31.0 (13.9)	.897

Values are presented as n (%) or mean ± SD. *MR*, Mitral regurgitation; *MS*, mitral stenosis; *LVEF*, left ventricular ejection fraction; *LVEDD*, left ventricular end diastolic diameter; *LV*, left ventricle; *LA*, left atrium; *sPAP*, systolic pulmonary arterial pressure.

## References

[1] Praz F., Borger M.A., Lanz J. (2025). 2025 ESC/EACTS guidelines for the management of valvular heart disease. Eur Heart J.

[2] Otto C.M., Nishimura R.A., Bonow R.O. (2021). 2020 ACC/AHA guideline for the management of patients with valvular heart disease: a report of the American College of Cardiology/American Heart Association Joint Committee on Clinical Practice Guidelines. Circulation.

[3] Pfannmueller B., Misfeld M., Verevkin A. (2021). Loop neochord versus leaflet resection techniques for minimally invasive mitral valve repair: long-term results. Eur J Cardiothorac Surg.

[4] Tomšič A., Holubec T., Sandoval E. (2024). Mitral valve repair with resection and non-resection techniques in Barlow's disease: a multi-center study. Int J Cardiol.

[5] Del Forno B., Tavana K., Ruffo C. (2023). Neochordae implantation versus leaflet resection in mitral valve posterior leaflet prolapse and dilated left ventricle: a propensity score matching comparison with long-term follow-up. Eur J Cardiothorac Surg.

[6] Ogami T., Chetkof E., Bonatti J.O. (2025). Posterior leaflet reconstruction in mitral valve repair: does resect versus respect strategy matter?. JTCVS Open.

[7] Choi J.W., Bishawi M., Zwischenberger B., Gaca J., Carr K., Glower D.D. (2024). Outcomes of leaflet resection vs chordal replacement for degenerative mitral regurgitation. Ann Thorac Surg.

[8] Kakuta T., Fukushima S., Minami K. (2023). What is the optimal mitral valve repair for isolated posterior leaflet prolapse to achieve long-term durability?. J Am Heart Assoc.

[9] Falk V., Seeburger J., Czesla M. (2008). How does the use of polytetrafluoroethylene neochordae for posterior mitral valve prolapse (loop technique) compare with leaflet resection? A prospective randomized trial. J Thorac Cardiovasc Surg.

[10] Kwon Y, Kim HJ, Kim JB (2025). Mitral valve repair with leaflet resection versus preservation for degenerative posterior leaflet prolapse. J Thorac Cardiovasc Surg.

[11] Zoghbi W.A., Adams D., Bonow R.O. (2017). Recommendations for noninvasive evaluation of native valvular regurgitation: a report from the American Society of Echocardiography developed in collaboration with the Society for Cardiovascular Magnetic Resonance. J Am Soc Echocardiogr.

[12] Lang M., Vitanova K., Voss B. (2023). Beyond the 10-year horizon: mitral valve repair solely with chordal replacement and annuloplasty. Ann Thorac Surg.

[13] Lange R., Guenther T., Noebauer C. (2010). Chordal replacement versus quadrangular resection for repair of isolated posterior mitral leaflet prolapse. Ann Thorac Surg.

[14] Carpentier A. (1983). Cardiac valve surgery--the "French correction". J Thorac Cardiovasc Surg.

[15] Mutsuga M., Narita Y., Tokuda Y. (2022). Predictors of failure of mitral valve repair using artificial chordae. Ann Thorac Surg.

[16] Park M.H., van Kampen A., Zhu Y. (2024). Neochordal goldilocks: analyzing the biomechanics of neochord length on papillary muscle forces suggests higher tolerance to shorter neochordae. J Thorac Cardiovasc Surg.

[17] Di Mauro M., Bonalumi G., Giambuzzi I. (2022). Mitral valve repair with artificial chords: tips and tricks. J Card Surg.

[18] von Oppell U.O., Mohr F.W. (2000). Chordal replacement for both minimally invasive and conventional mitral valve surgery using premeasured Gore-Tex loops. Ann Thorac Surg.

[19] David T.E. (2025). Replacement of chordae tendineae with expanded polytetrafluoroethylene sutures. Ann Thorac Surg.

[20] Suri R.M., Clavel M.A., Schaff H.V. (2016). Effect of recurrent mitral regurgitation following degenerative mitral valve repair: long-term analysis of competing outcomes. J Am Coll Cardiol.

